# LncRNA MIR503HG regulates NETs‐mediated NLRP3 inflammasome activation and NSCLC metastasis by enhancing the ubiquitination of C/EBPβ

**DOI:** 10.1002/ctm2.70342

**Published:** 2025-06-09

**Authors:** Xin Ye, Chen Fang, Weiwei Hong, Xiaoying Qian, Biao Yu, Bingbiao Zhou, Xinyuan Yao, Dengying Chen, Chengsi Shu, Chuanhong Luo, Yong Wang, Yong Li

**Affiliations:** ^1^ Department of Medical Oncology The First Affiliated Hospital of Nanchang University Nanchang China; ^2^ Medical Innovation Center The First Affiliated Hospital of Nanchang University Nanchang China

**Keywords:** C/EBPβ, DNA methylation, MIR503HG, NETs, NLRP3, RNF43, ubiquitination

## Abstract

**Background:**

Neutrophil extracellular traps (NETs) are pivotal in the metastasis of non‐small cell lung cancer (NSCLC). Our previous research demonstrated that NETs facilitate NSCLC metastasis by triggering the stimulation of the NOD‐like receptor protein 3 (NLRP3) inflammasome, which is mediated through the suppression of the long non‐coding RNA MIR503HG. However, the precise molecular mechanisms linking MIR503HG to NLRP3 are still not fully understood.

**Methods:**

By employing protein mass spectrometry and the Human TFDB database, key molecules involved in NLRP3 regulation were identified. The involvement of CCAAT enhancer binding protein beta (C/EBPβ) in NSCLC metastasis was examined in both cellular and animal models. Dual‐luciferase and CUT&RUN assays confirmed the mechanism by which C/EBPβ controls NLRP3. The regulatory relationship between MIR503HG and C/EBPβ was explored through RNA pulldown, RNA immunoprecipitation and coimmunoprecipitation assays. Additionally, methylation‐specific PCR and other studies revealed that NETs suppress MIR503HG via DNA methylation.

**Results:**

We found that C/EBPβ mediates the regulation of NLRP3 by MIR503HG. Further investigation confirmed that C/EBPβ promotes the migration and invasion of NSCLC both in vivo and in vitro and is highly expressed in NSCLC tissue. Mechanistically, C/EBPβ binds to the NLRP3 promoter to promote NLRP3 expression. Conversely, MIR503HG suppressed C/EBPβ expression by facilitating C/EBPβ interaction with the E3 ubiquitin ligase RNF43, which in turn reduced NLRP3 expression and NSCLC metastasis. Meanwhile, we investigated the mechanism by which NETs inhibit MIR503HG expression and found that DNA methylation is involved in the suppression of MIR503HG by NETs. Additionally, reversing this methylation partially restored MIR503HG and NLRP3 expression and mitigated the metastatic effects of NETs in NSCLC.

**Conclusions:**

This study emphasises the critical roles of C/EBPβ and DNA methylation in NETs‐mediated NSCLC metastasis. These findings unveil C/EBPβ and DNA methylation as potential novel targets for NSCLC with high NETs expression.

**Key points:**

- NETs suppress the expression of MIR503HG by inducing promoter DNA methylation.
- C/EBPβ binds to the NLRP3 promoter to promote NLRP3 expression.
- MIR503HG inhibits the expression of C/EBPβ protein by promoting the interaction between C/EBPβ and the E3 ubiquitin ligase RNF43, thereby repressing NLRP3 expression.

## Graphical abstract

1. NETs suppress the expression of MIR503HG by inducing promoter DNA methylation.
2. C/EBPβ binds to the NLRP3 promoter to promote NLRP3 expression.
3. MIR503HG inhibits the expression of C/EBPβ protein by promoting the interaction between C/EBPβ and the E3 ubiquitin ligase RNF43, thereby repressing NLRP3 expression.

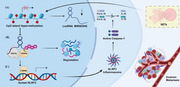

## 1 INTRODUCTION

Non‐small cell lung cancer (NSCLC) represents a leading cause of mortality related to cancer globally. Despite advancements in targeted and immunotherapy treatments that have improved survival rates, the overall survival rate at 5 years remains only 20–30%.^1^ This phenomenon is due primarily to high recurrence and metastasis rates following treatment.^2^ Thus, understanding the precise mechanisms of NSCLC metastasis and identifying new therapeutic targets are crucial.

Neutrophil extracellular traps (NETs) consist of a network formed by DNA, histones and antimicrobial proteins, which are released by activated neutrophils. Their function to capture and remove extracellular pathogens, playing a key role in antimicrobial defense.^3^ ^,4^ Recent studies have increasingly linked NETs to tumour metastasis.^5^ ^–^ ^7^ For example, research by Xia et al.^8^ demonstrated that NETs formation can enhance postoperative metastasis in gastric cancer patients. Additionally, our findings indicate that NETs facilitate NSCLC metastasis by activating the NLRP3 inflammasome pathway through the suppression of the MIR503HG.^9^ However, the precise interaction between MIR503HG and NLRP3 ambiguous.

Long non‐coding RNAs (lncRNAs) are RNA molecules exceeding 200 nucleotides in length that are essential for gene regulation, chromatin modification and cell cycle control.^10^ ^11^ Although lncRNAs lack the ability to encode proteins, they exert their functions by regulating various proteins. For example, the lncRNA CRLM1 has been implicated in bowel cancer metastasis, with its regulation of heterogeneous nuclear ribonucleoprotein K (hnRNPK) shown to slow tumour growth and reduce metastasis.^12^ Similarly, it has been documented that the lncRNA VAL promote metastasis by inhibiting Trim16‐mediated degradation of vimentin.^13^ Building on these observations, we proposed the hypothesis that MIR503HG may suppress NLRP3 expression by modulating specific key molecules that remain to be identified.

In this research, we observed that C/EBPβ is crucial for the regulation of NLRP3 by MIR503HG. Specifically, MIR503HG inhibits NLRP3 expression by promoting RNF43‐mediated ubiquitination and degradation of C/EBPβ. Furthermore, our investigation into the low expression of MIR503HG under NETs stimulation revealed that NETs induce the down‐regulation of MIR503HG via DNA methylation. These findings reveal a novel mechanism linking NETs to NSCLC metastasis.

## 2 METHODS

### 2.1 Clinical samples and cell sources

Human tissue specimens utilised in this study were procured through the Pathology Division at Nanchang University's Primary Teaching Hospital. NSCLC cellular models originated from the authenticated repository maintained by the Chinese Academy of Sciences Cell Resource Center (Shanghai, China). Cellular propagation was conducted in DMEM (Solarbio, Beijing, China) containing 10% foetal bovine serum (FBS; Gibco, USA), to ensure optimal growth conditions for the experimental procedures.

### 2.2 Extraction of neutrophils

Neutrophils were isolated using a human peripheral blood neutrophil isolation kit (Solarbio). Placed in RPMI 1640 (BI, Israel), which was fortified with 10% FBS to sustain their growth and vitality. To ensure the quality of the isolation process, the purity of neutrophils, exceeding 98%, and their viability, maintained above 95%, were rigorously evaluated through the implementation of Giemsa staining and trypan blue exclusion assays.

### 2.3 Extraction of NETs

Neutrophils were exposed to phorbol‐12‐myristate‐13‐acetate (Sigma, USA) for a duration of 4 h to stimulate the generation of NETs. Next, NETs were collected using a multistep centrifugation procedure as outlined in previous studies.^14^ Following the NETs formation, the supernatants enriched with NETs components underwent centrifugation for additional purification, then preserved at −80°C.

### 2.4 Methylation‐specific PCR

After incubating the cells with NETs for 12 h, DNA was extracted from samples. MSP primers were designed using the MethPrimer website. The DNA was subjected to bisulphite conversion using the EZ DNA Methylation‐Gold™ Kit (Zymo Research, USA). Next, a methylation‐specific PCR (MSP‐PCR) mixture was created with the ZymoTaq™ PreMix Kit (Zymo Research). The amplification products were evaluated through agarose gel electrophoresis.

### 2.5 RNA pulldown assays

A plasmid designed for MIR503HG overexpression served as the template to amplify DNA fragments corresponding to either the sense or antisense strand of MIR503HG. In vitro transcription of either the sense or antisense RNA was carried out using the DNA template, with the RNA being labelled by Biotin RNA Labeling Mix (Roche). Subsequently, 5 mg of the protein sample was mixed with 3 µg of biotinylated RNA and then incubated at 4°C in the dark for a duration of 1 h. Beads that had been washed were incorporated into the mixture, which was subsequently incubated overnight in the dark at 4°C. Following five washes with 1 mL of NT2 buffer, the sample was combined with sodium dodecyl sulfate (SDS) loading buffer and subsequently heat block for 10 min. The protein samples were subsequently subjected to sodium dodecyl sulfate ‐ polyacrylamide gel electrophoresis (SDS‐PAGE) and stained with silver using a Fast Silver Stain Kit (Beyotime). The gel strips were then excised for further analysis, including mass spectrometry or Western blotting, to detect the target proteins.

### 2.6 RNA immunoprecipitation assays

According to the provided protocol, the RNA Immunoprecipitation (RIP) kit (Bersinbio, China) was used for the experiment. Immunoprecipitation was performed with anti‐Flag antibodies, and after RNA elution, the samples were analysed via RT‐qPCR.

### 2.7 Luciferase assay

Genomic DNA was isolated from A549 cells. Amplified the promoter region of NLRP3 from it and inserted it into the PGL4.2 vector. Subsequently, co‐transfected the C/EBPβ, NLRP3 promoter or reninase plasmids. Luciferase activity was measured using the Dual‐Luciferase Reporter Assay Kit (Promega, Madison, WI, USA).

### 2.8 Cleavage under targets and release using nuclease (CUT&RUN)

Using the Hyperactive pG‐MNase CUT&RUN Assay Kit (Vazyme, China), the procedure was performed following the instructions provided in the protocol. Immunoprecipitation was carried out with anti‐Flag antibodies and analysed via RT‒qPCR.

### 2.9 Coimmunoprecipitation

Experiments were conducted 48 h after transfection of the relevant plasmids into the cells. The cells were lysed with lysis buffer (Servicebio, Wuhan, China) that included protease inhibitors. The lysates were incubated overnight at 4°C with specific antibodies while being gently agitated. Subsequently, protein A or G beads (Santa Cruz Biotechnology) were introduced into the solution and allowed to sit at 4°C for an extra 4–6 h. The beads were subjected to a minimum of three washes. The immunoprecipitation results were analysed by Western blotting.

### 2.10 GST pulldown assays

GST‐tagged C/EBPβ and His‐tagged RNF43 were expressed in BL21 chemically competent cells (TransGen Biotech, China) and purified in vitro. Bacteria were lysed using sonication. Glutathione magnetic agarose beads were incubated with GST‐C/EBPβ or GST‐only lysates for 4 h at 4°C with gentle shaking. Subsequently, purified His‐RNF43 proteins, obtained using HisSep Ni‐NTA MagBeads (Yesen, China), were introduced into the solution and allowed to incubate overnight at 4°C with gentle agitation. The presence of His‐RNF43 was detected using anti‐His antibodies.

### 2.11 Immunofluorescence

Cellular samples underwent initial immobilisation in fixative solution (20 min, RT) followed by three PBS washing cycles (5 min each). Membrane permeabilisation was achieved through 0.5% Triton X‐100 treatment (15 min, RT), succeeded by 1‐h incubation in 5% normal goat serum blocking buffer. Immunostaining commenced with primary antibody application: rabbit anti‐C/EBPβ (23431‐1‐AP, 1:250; ProteinTech, Wuhan, China) and mouse anti‐Flag (F1804, 1:500; Merck, Darmstadt, Germany) under overnight refrigeration (4°C). Then, add the suitable secondary antibody and DAPI (Beyotime, China). Images were acquired utilising both a fluorescence microscope (Zeiss) and a confocal laser scanning microscope (Olympus).

### 2.12 Immunohistochemistry

Tissue was sectioned into 5 mm thick slices and subjected to immunohistochemical analysis.^15^ Tissue sections were first heated in EDTA antigen retrieval solution (pH = 9.0) and then natural cooling. Subsequently, they were treated with 3% hydrogen peroxide for 10 min. After a 20‐min blocking with goat serum, treated with primary antibodies—anti‐C/EBPβ (1:250; ProteinTech; 23431‐1‐AP) and anti‐RNF43 (1:500; CSB‐PA019892LA01HU; CUSABIO, China). These antibodies were incubated with the sections overnight at 4°C. Two blinded assessors independently evaluated the expression levels of C/EBPβ and RNF43. Immunostaining intensity (graded 0–3: 0 = absent, 1 = mild, 2 = intermediate, 3 = marked); cellular positivity proportion (categorised 0–4: 0 = 0%, 1 = ≤25%, 2 = 26‐50%, 3 = 51‐75%, 4 = ≥76%). The histopathological score was subsequently determined through multiplication of the intensity grade and cellular positivity category values.

### 2.13 Transient transfection

The cells were seeded at appropriate densities in Petri dishes in advance. Plasmids or siRNAs were transfected using Lipo8000™ Transfection Reagent (Beyotime). The expression quantities of the target molecules were evaluated using Western blotting or RT‒qPCR after 24‒48 h.

The C/EBPβ overexpression plasmid pENTER‐C/EBPβ and its corresponding control plasmid pENTER were sourced from Vigene Biosciences (Shandong, China). The MIR503HG overexpression plasmid (with control GV208) and the C/EBPβ knockdown plasmid (with control GV248) were obtained from Shanghai GeneChem Co., Ltd. RNF43 overexpression and knockdown plasmids were obtained from MiaoLingPlasmid (Wuhan, China). All siRNAs were synthesised by Suzhou GenePharma Co., Ltd. All sequences are listed in Table S1. Note that sh C/EBPβ and sh RNF43 represent plasmids that function to knock down the corresponding genes. Additionally, sh1, sh2, si NC, si1 and si2 represent distinct knockdown cell clones, with si NC defined as si negative controls’.

### 2.14 Transwell invasion assay

A 24‐well Transwell plate was used, with 100 µL of 5% Matrigel (Corning, USA) applied to the upper chamber. Cellular suspensions containing 6 × 10^4^ viable cells in 200 µL serum‐deprived DMEM were seeded in upper compartments, while lower chambers contained 700 µL complete medium generating chemotactic gradient (20% FBS). After 48 h of incubation, fix with 4% paraformaldehyde. Then proceed with the staining. Quantitative analysis was conducted by randomly selecting five fields under an optical microscope. Subsequently, the data were analysed using ImageJ software.

### 2.15 Wound healing assay

Culture the cells in six‐well plates. Employing the tip of a 200 µL pipette to create wounds, ensuring that the scratch width remained uniform throughout. Following three washes with PBS to eliminate any debris, then incubated in a medium containing 1% serum for incubation. Images were captured and wound widths were evaluated using ImageJ software.

### 2.16 Ubiquitylation assay

The plasmids were transfected for 48 h, followed by a 6‐h treatment with MG132. One‐tenth of the lysates from the cells were reserved as a total protein sample, while the remainder were used for the ubiquitination assays. The cells were subjected to lysis using Buffer I. Ni‐NTA beads (Takara) were subsequently introduced and mixed with gentle rotation at room temperature for 4 h to facilitate the binding of His‐tagged proteins. The proteins were subjected to a washing procedure, first with Buffer I and then with Buffer II, performing three washes with each. Following these washes, ubiquitination levels in the target proteins were evaluated through Western blot analysis. The preparation details for Buffer I and Buffer II solutions are provided in Table S2.

### 2.17 In vivo lung metastasis experiment

Immunocompromised murine models (BALB/cAnN.Cg‐Foxn1nu, male, 4‐week‐old) were procured from a certified vendor (Hangzhou Ziyuan Laboratory Animal Co., Zhejiang, China) under SPF conditions. A549 adenocarcinoma cells harbouring stable C/EBPβ‐knockdown and scramble control counterparts were preconditioned with NETs. Cellular suspensions (2 × 10^6^ cells/200 µL PBS) were aseptically administered via lateral tail vein injection. The cells were injected into the body via the tail vein. After 8 weeks, the lungs were harvested and subjected to histological analysis using H&E staining.

### 2.18 RT‐qPCR

RNA isolation was performed with TRIZOL reagent (Servicebio) following standard protocols. The cDNA synthesis was subsequently conducted employing the M5 Sprint qPCR RT Kit (Mei5bio, China) according to the manufacturer's specifications. Amplification of cDNA targets was carried out via RT‐qPCR using PerfectStart Green qPCR SuperMix (TransGen Biotech). Primer pair sequences utilised in this study are detailed in Table S3.

### 2.19 Western blotting

Lysis of samples was performed using RIPA buffer (Beyotime) that included protease inhibitors. The protein extracts were separated by SDS‐PAGE. Subsequently, the samples were incubated with the primary antibody at 4°C for an overnight period.

Detection was carried out using a chemiluminescence (ECL) detection kit (ProteinTech) on a chemiluminescence imaging system (ChemiDoc). Antibody information is provided in Table S4.

### 2.20 Databases

Transcription factors for NLRP3 were predicted using the Human TFDB database (http://bioinfo.life.hust.edu.cn/HumanTFDB#!/). Methylation levels in tumour and adjacent tissues were assessed using MEXPRESS (https://mexpress.be/).^16^ Additionally, the correlation between C/EBPβ and prognostic indicators of various pathological types of NSCLC, as well as its association with NLEPP3 expression, was analysed using the GEPIA database (http://gepia.cancer‐pku.cn/). The structures of C/EBPβ and RNF43 were predicted using I‐TASSER (https://zhanggroup.org/I‐TASSER‐MTD/).

### 2.21 Statistical analysis

ImageJ and GraphPad Prism 8 were employed for data analysis. Student's *t*‐test compared two groups, while one‐way ANOVA assessed multiple groups. Data are shown as mean ± SD unless otherwise noted. Statistical significance was defined as *p* < .05.

## 3 RESULTS

### 3.1 Identification of C/EBPβ as a key molecule that binds MIR503HG and regulates NLRP3

**FIGURE 1:**
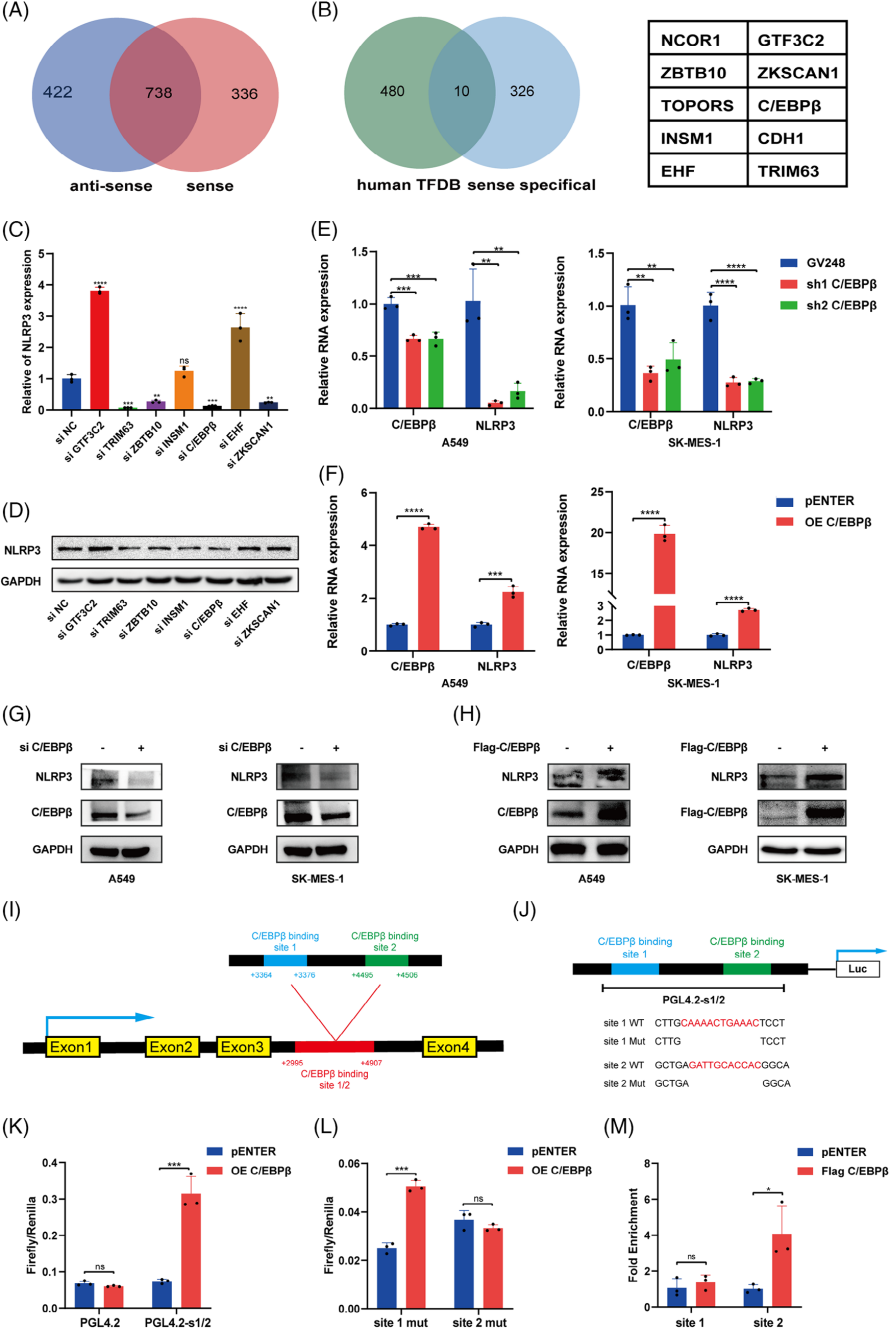
Identification of C/EBPβ as a transcription factor that binds MIR503HG and regulates NLRP3. (A) Protein mass spectrometry was used to analyse proteins bound to the MIR503HG sense and antisense strands. (B) Comparison of the predicted transcription factors of NLRP3 with proteins specifically bound to the MIR503HG‐sense strand. (C and D) RT‒qPCR and Western blot analyses were conducted to measure the NLRP3 mRNA and protein levels following the down‐regulation of seven transcription factors in A549 cells. (E and F) RT‐qPCR was used to detect the mRNA levels of the NLRP3 gene following knockdown or overexpression of C/EBPβ. (G and H) Western blot analysis was employed to measure the protein levels of the NLRP3 gene following knockdown or overexpression of C/EBPβ. A schematic diagram illustrating the predicted binding sites (I) and mutation patterns (J) of C/EBPβ on the NLRP3 promoter. (K and L) Luciferase assays were conducted to assess luciferase activity resulting from C/EBPβ overexpression. (M) CUT&RUN assay was used to confirm that C/EBPβ binds to the NLRP3 promoter. Data in (C, E‐F, K–M) are the mean ± SD of three independent experiments. The statistical method used for the data in (F, K–M) is Student's *t*‐test, while the data in (C and E) were analysed using one‐way analysis of variance (ANOVA). ns indicates no statistical significance, *p < .05, **p < .01, ***p < .001, ****p < .0001.

In our previous study, we established that MIR503HG modulates NLRP3 expression through transcriptional regulation. We hypothesised that certain transcription factors mediate this effect. To identify key molecules, we used protein mass spectrometry to identify proteins specifically binding to MIR503HG (Figure 1A). A comparison of these proteins with predicted NLRP3 transcription factors led us to identify 10 candidates (Figure 1B). However, there are reports that NCOR1, TOPORS and CDH1 play inhibitory roles in tumour progression^17^, ^18^, ^19^; therefore, we believe that the remaining seven genes may be involved in the regulation of NLRP3. We tested the effects of knocking down these candidate proteins on NLRP3 expression. The results showed that knocking down C/EBPβ, TRIM63, ZBTB10 and ZKSCAN1 significantly decreased NLRP3 mRNA (Figure 1C) and protein expression (Figure 1D). Given that C/EBPβ influences nephritis through NLRP3,^20^ we propose the hypothesis that C/EBPβ acts as a crucial regulator of NLRP3 within the framework of MIR503HG.

TCGA database evaluation indicated that elevated expression of C/EBPβ is markedly linked to unfavourable prognosis in patients with NSCLC (Figure S1A–F). Correlation analysis revealed a positive link between C/EBPβ and NLRP3 expression (Figure S1G–I), aligning with our hypothesis. In addition, C/EBPβ knockdown resulted in decreased NLRP3 mRNA (Figure 1E) and protein levels (Figure 1G), while C/EBPβ overexpression produced the opposite effects (Figure 1F,H). To further explore the mechanism by which C/EBPβ promotes NLRP3 expression, we used the Human TFDB database to predict C/EBPβ binding sites on the NLRP3 promoter, identifying two high‐scoring sites, site 1 and site 2. These sites were incorporated into a luciferase reporter vector. A luciferase assay revealed that C/EBPβ overexpression significantly enhanced luciferase activity in this promoter region (Figure 1K). When we individually deleted site 1 or site 2, C/EBPβ continued to activate the promoter after site 1 deletion but not after site 2 deletion (Figure 1L). Furthermore, CUT&RUN has confirmed that C/EBPβ binds to the region where site2 is located, rather than site1 (Figure 1M). In summary, these findings demonstrate that C/EBPβ acts as a transcriptional regulator to promote NLRP3 expression and may be involved in the regulation of NLRP3 by MIR503HG.

### 3.2 C/EBPβ enhances the metastasis of NSCLC and is involved in the inhibition process mediated by MIR503HG

**FIGURE 2:**
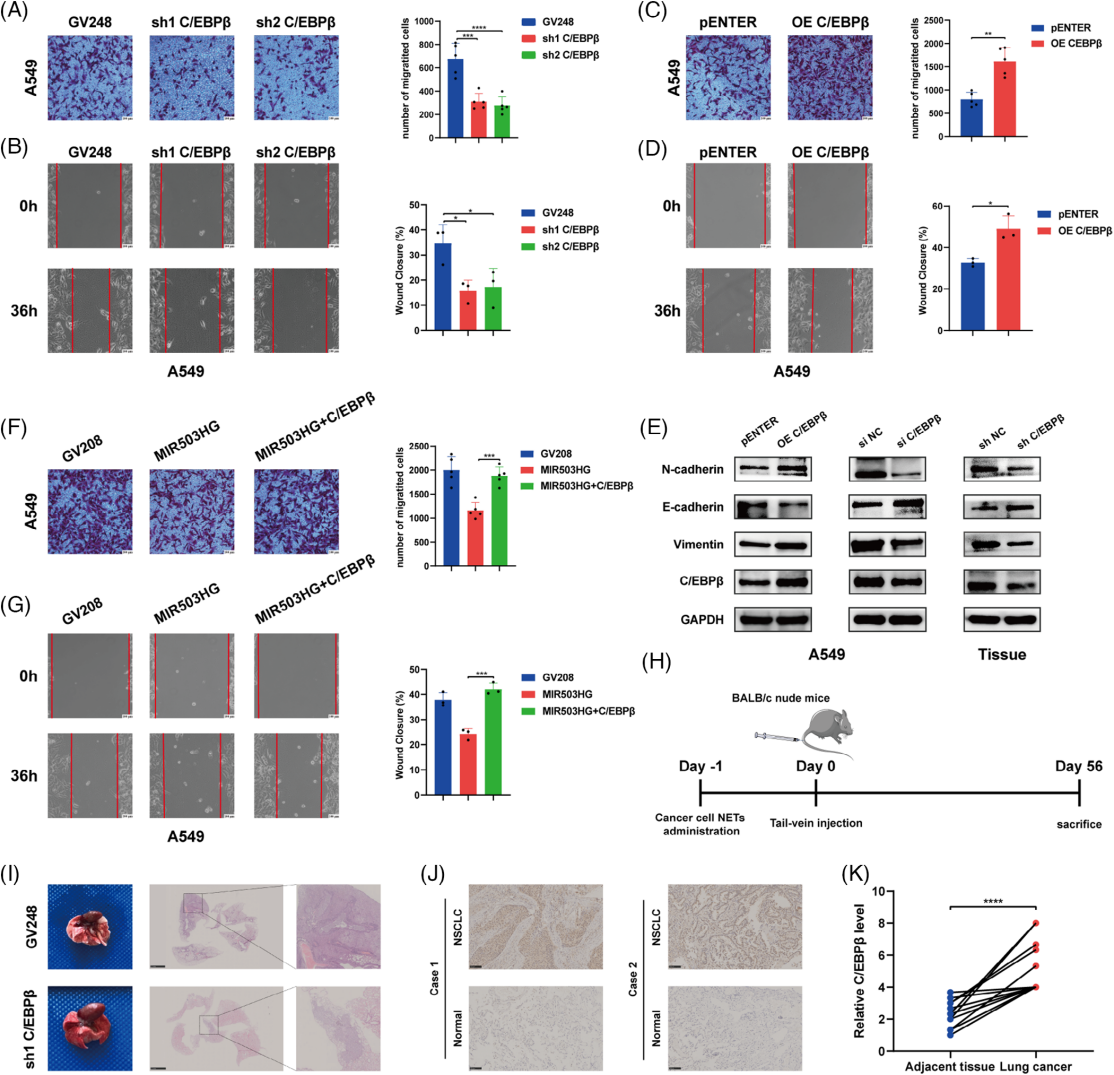
C/EBPβ enhances the metastasis of NSCLC cells both in vivo and in vitro following NETs stimulation. (A and B) The invasive potential and migratory capacity of NSCLC cells following C/EBPβ down‐regulation were assessed using transwell and wound‐healing assays; scale bar, 100 µm. (C and D) These assays were repeated to evaluate the effects of C/EBPβ overexpression on cell invasion and migration. (E) Western blot analysis was conducted to assess the expression of EMT markers (N‐cadherin, E‐cadherin and vimentin) in NSCLC cells following overexpression or knockdown of C/EBPβ, as well as in tissues post C/EBPβ knockdown. (F and G) The impact of MIR503HG overexpression alongside C/EBPβ activation on NSCLC cell invasion and migration was analysed. (H) A schematic diagram of in vivo experiments used to investigate the role of C/EBPβ in NETs‐induced metastasis. (I) Representative images of lung specimens, including gross morphology and H&E staining of metastatic nodules in mice. (J and K) Representative data and a summary from 17 paired samples showing C/EBPβ expression levels in NSCLC tissues compared with adjacent normal tissues. Each data point represents the average value derived from two independent blinded assessors. Statistical analysis was performed using paired *t* tests; scale bar, 100 µm. The data in (A–D, F and G) are presented as mean ± SD. Data in (A, C and F) were derived from five independent experiments, while data in (B, D and G) were obtained from three independent experiments. The statistical method used for the data in (C and D) is Student's *t*‐test, while the data in (A, B, F and G) were analysed using one‐way analysis of variance (ANOVA). *p < .05, **p < .01, ***p < .001, ****p < .0001.

To explore the function of C/EBPβ in promoting the metastasis of NSCLC through NETs, we first conducted invasion and wound healing assays. These assays revealed reduced invasion (Figures 2A and S2A) and migration (Figures 2B and S2B) in NSCLC cells following C/EBPβ knockdown. Conversely, the overexpression of C/EBPβ via plasmid transfection significantly increased both the invasion (Figures 2C and S2C) and migration (Figures 2D and S2D) of NSCLC cells. Moreover, we assessed the function of C/EBPβ in the epithelial‒mesenchymal transition (EMT). C/EBPβ overexpression led to decreased E‐cadherin levels and increased N‐cadherin and vimentin expression, whereas C/EBPβ knockdown produced the opposite effects (Figures 2E and S2E). Additionally, transfection of C/EBPβ in cells overexpressing MIR503HG attenuated the suppressive impact of MIR503HG on NSCLC cell activity (Figures 2F,G and S2F,G), indicating that C/EBPβ might participate in the regulatory processes that are mediated by MIR503HG.

These in vitro results demonstrate that C/EBPβ promotes NSCLC metastasis. To achieve a more comprehensive understanding of the in vivo function of C/EBPβ, we generated C/EBPβ‐knockdown and control NSCLC cells, which were administered to mice through the tail vein after 12 h of NETs treatment. After 8 weeks, the mice were euthanised humanely, and lung tumour size was demonstrated using H&E staining (Figure 2I). The data showed that C/EBPβ knockdown markedly decreased NETs‐induced metastasis in vivo. Next, we harvested the lungs from the previous A549 lung metastasis model and extracted the lung tumour tissues formed by A549. Analysis of C/EBPβ and EMT‐related proteins revealed results consistent with our in vitro experimental findings (Figure 2E). Additionally, immunohistochemical analysis of 17 pairs of NSCLC tissues and their adjacent normal tissues revealed elevated C/EBPβ expression in NSCLC tissues (Figure 2J,K), corroborating our findings. These findings collectively indicate that C/EBPβ enhances the metastasis of NSCLC cells and is involved in the inhibition process mediated by MIR503HG.

### 3.3 MIR503HG binds to C/EBPβ and facilitates its degradation through the ubiquitin pathway

**FIGURE 3:**
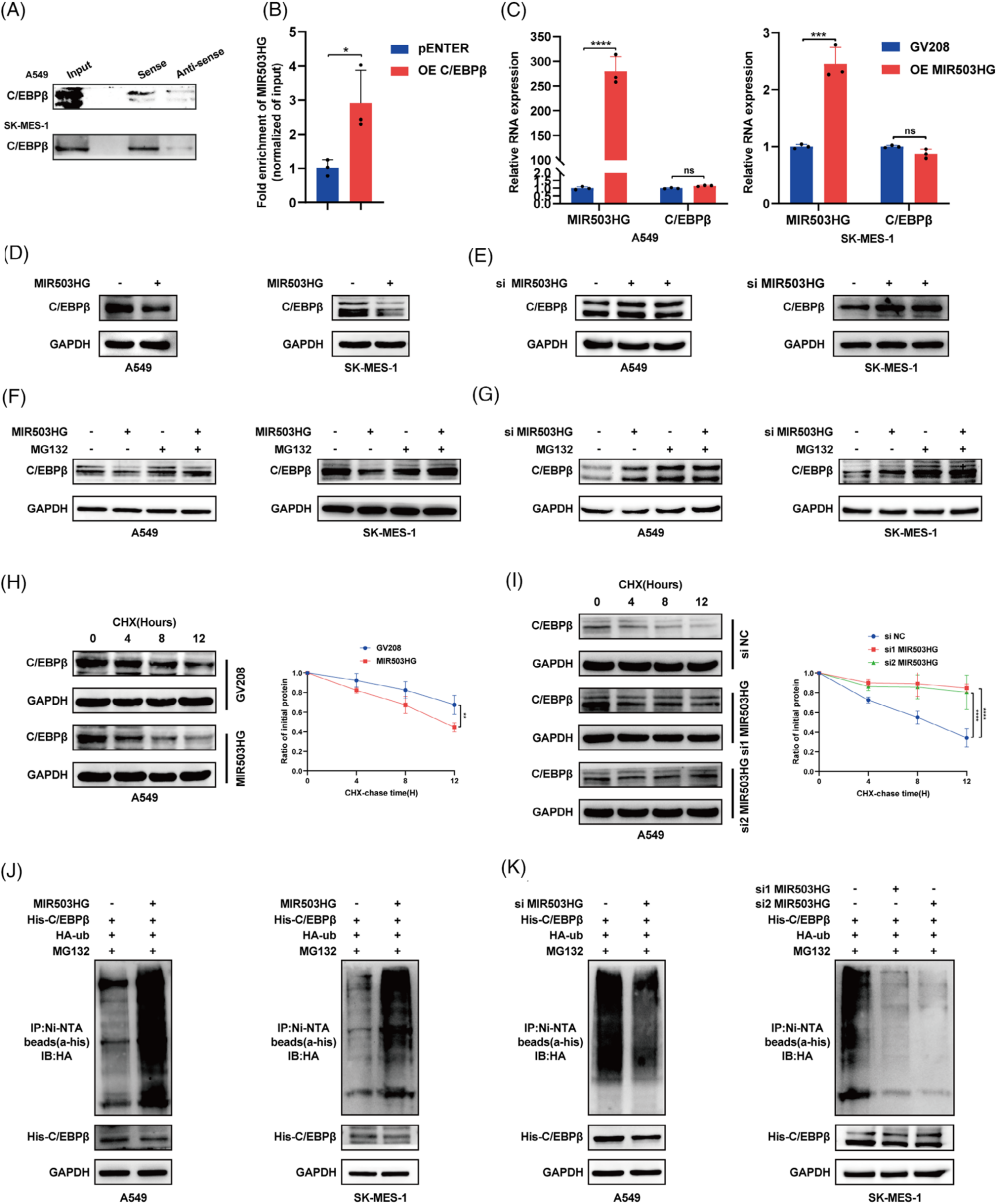
MIR503HG binds to C/EBPβ and facilitates its degradation through the ubiquitin pathway. (A) RNA pulldown and (B) RIP assays confirmed the interaction between MIR503HG and C/EBPβ. (C) RT‒qPCR analysis was performed to assess C/EBPβ mRNA levels in NSCLC cells following MIR503HG overexpression. Western blot analyses were used to detect C/EBPβ protein expression after MIR503HG overexpression (D) or knockdown (E). Additionally, C/EBPβ protein levels were evaluated by Western blot following MIR503HG knockdown or overexpression and treatment with MG132 (F and G) or cycloheximide (CHX) (H and I). Western blotting was also used to measure C/EBPβ ubiquitination levels after MIR503HG overexpression (J) or knockdown (K). Data in (B, C, H and I) are the mean ± SD. of three independent experiments. The statistical method used for the data in (B and C) is Student's *t*‐test, while the data in (H and I) were analysed using two‐way analysis of variance (ANOVA). ns indicates no statistical significance, *p < .05, **p < .01, ***p < .001, ****p < .0001.

To clarify the role of MIR503HG in inhibiting NLRP3 expression via C/EBPβ regulation, we first demonstrated the interaction between MIR503HG and C/EBPβ through RNA pulldown (Figure 3A) and RIP (Figure 3B) assays. We then assessed the effect of MIR503HG modulation on C/EBPβ expression. Our findings indicated that overexpression of MIR503HG has no influence on C/EBPβ mRNA levels (Figure 3C), but significantly reduces C/EBPβ protein levels (Figure 3D). As expected, the knockdown of the MIR503HG gene resulted in a rise in C/EBPβ protein levels (Figure 3E). These findings preliminarily establish that MIR503HG represses NLRP3 expression by modulating C/EBPβ.

However, the exact process through which MIR503HG suppresses C/EBPβ remains unknown. Previous research has shown that lncRNAs can modulate the expression of RNA‐binding proteins via the ubiquitination pathway.^21^, ^22^ Interestingly, the repression of C/EBPβ by MIR503HG was reversed following the administration of the proteasome inhibitor MG132 (Figure 3F,G). Furthermore, upon inhibition of protein synthesis using cycloheximide (CHX), it was found that high levels of MIR503HG decreased the half‐life of C/EBPβ (Figures 3H and S3A), whereas MIR503HG knockdown prolonged it (Figures 3I and S3B). Finally, we investigated whether the degradation of C/EBPβ by MIR503HG is mediated through ubiquitination. We detected a notable increase in the ubiquitin signal of C/EBPβ after MIR503HG overexpression (Figure 3J), which was reversed following MIR503HG knockdown (Figure 3K). These findings collectively suggest that MIR503HG inhibits the expression of C/EBPβ through the ubiquitination pathway, thereby suppressing NLRP3 expression.

### 3.4 RNF43 is an E3 ubiquitin ligase for C/EBPβ, interacting with C/EBPβ to promote its degradation

**FIGURE 4:**
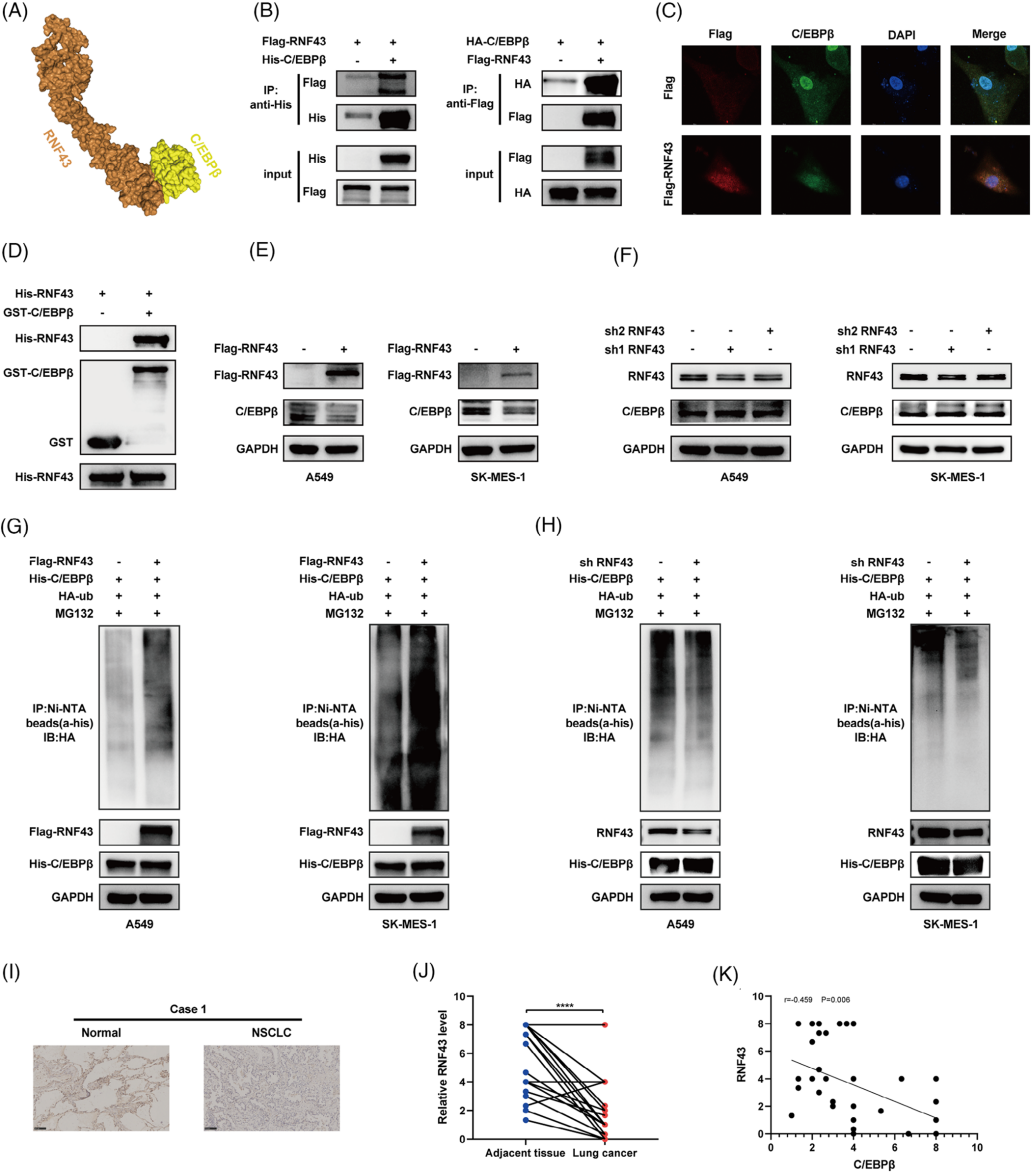
RNF43 is an E3 ubiquitin ligase for C/EBPβ, interacting with C/EBPβ to promote its degradation. (A) Molecular docking of C/EBPβ and RNF43 SLUG. (B) Coimmunoprecipitation was used to analyse the interaction between RNF43 and C/EBPβ. (C) Confocal microscopy revealed the colocalisation of C/EBPβ (red) and RNF43 (green) in indicator cells. (D) GST pulldown assays confirmed the direct interaction between RNF43 and C/EBPβ. Western blot analysis was performed to measure C/EBPβ protein expression following RNF43 overexpression (E) or knockdown (F). Additionally, the ubiquitination level of C/EBPβ was assessed by Western blot after RNF43 overexpression (G) or knockdown (H). (I and J) Representative images and summary data from 17 paired samples illustrating RNF43 expression in NSCLC tissues compared with adjacent normal tissues. Each data point represents the average value derived from two independent blinded assessors. Statistical analysis was conducted using paired *t* tests; scale bar, 100 µm. ****p < .0001. (K) Correlation analysis of C/EBPβ and RNF43 expression levels in tissues.

MIR503HG is not a ubiquitin ligase itself. Thus, we aimed to identify the ubiquitin ligases involved in regulating C/EBPβ. Previous studies have shown that lncRNAs can inhibit RNA‐binding proteins by facilitating their interaction with E3 ubiquitin ligases.^23^ Using mass spectrometry of MIR503HG, we identified several E3 ligases, with RNF43 being the most likely mediator of the regulation of C/EBPβ by MIR503HG. This observation is backed by earlier studies indicating that RNF43 and C/EBPβ both play roles in the Wnt/β‐catenin pathway,^24^, ^25^ and predictive models suggest a high probability of their interaction (Figure 4A). Coimmunoprecipitation (Figure 4B) and immunofluorescence confocal microscopy (Figure 4C) confirmed the interaction between C/EBPβ and RNF43. GST pulldown assays further verified their direct binding (Figure 4D). As anticipated, overexpression of RNF43 resulted in a reduction of the levels of C/EBPβ protein (Figure 4E), whereas the knockdown of RNF43 had the opposite effect (Figure 4F). The influence of RNF43 on C/EBPβ was nullified upon MG132 treatment (Figure S4A,B). Additionally, CHX assays demonstrated that RNF43 overexpression shortened the half‐life of C/EBPβ (Figure S4C), whereas RNF43 knockdown extended it (Figure S4D). We then assessed C/EBPβ ubiquitination after manipulating RNF43 levels and found that RNF43 overexpression increased C/EBPβ ubiquitination (Figure 4G), whereas RNF43 knockdown had the opposite effect (Figure 4H). Clinically, RNF43 expression is significantly reduced in NSCLC tissues (Figure 4I,J). Furthermore, our analysis showed an inverse relationship between C/EBPβ and RNF43 expression (Figure 4K), reinforcing our findings. These results strongly suggest that RNF43 is an E3 ubiquitin ligase for C/EBPβ, interacting with C/EBPβ to promote its degradation.

### 3.5 MIR503HG acts as a scaffold to facilitate the binding of C/EBPβ to RNF43

**FIGURE 5:**
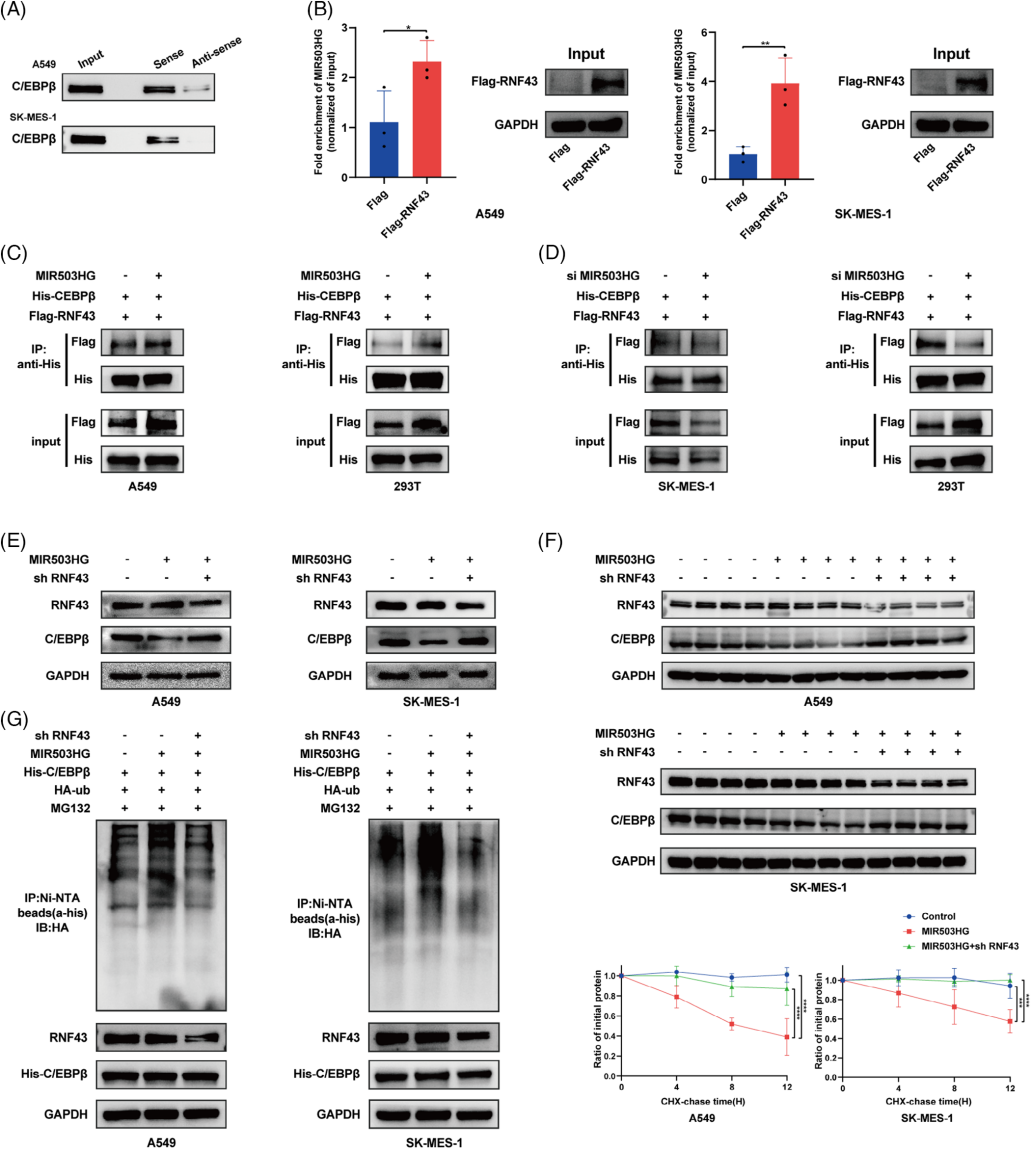
MIR503HG acts as a scaffold to facilitate the binding of C/EBPβ to RNF43. RNA pulldown (A) and RIP (B) assays confirmed the binding of MIR503HG to RNF43. Immunoprecipitation was utilised to assess the interaction between C/EBPβ and RNF43 upon MIR503HG overexpression (C) or down‐regulation (D). After the cells were transfected with the aforementioned plasmids, Western blot analysis was conducted to evaluate the C/EBPβ protein levels in the untreated cells (E) and following CHX treatment (F). (G) Western blotting was also performed to determine the ubiquitination status of C/EBPβ in cells transfected with the plasmids. Data in (B and F) are the mean ± SD. of three independent experiments. The statistical method used for the data in (B) is Student's *t*‐test, while the data in (F) were analysed using two‐way analysis of variance (ANOVA). *p < .05, **p < .01, ***p < .001, ****p < .0001.

To demonstrate that MIR503HG enhances C/EBPβ degradation by promoting the interaction of C/EBPβ with RNF43, we first confirmed the binding of MIR503HG to RNF43 through RNA pull‐down and RIP assays. The results indicated successful pulldown of RNF43 by MIR503HG (Figure 5A) and notable enrichment of MIR503HG in RNA‒protein complexes captured with an anti‐RNF43 antibody (Figure 5B). We then assessed whether MIR503HG influences the binding of C/EBPβ to RNF43. The amount of RNF43 coprecipitated with C/EBPβ in MIR503HG‐overexpressing cells was greater (Figure 5C). Conversely, MIR503HG knockdown resulted in decreased RNF43 coprecipitation with C/EBPβ (Figure 5D). Additionally, to provide additional evidence for the function of RNF43 in the MIR503HG‐modulated control of C/EBPβ, we overexpressed MIR503HG in RNF43‐knockdown cells and analysed C/EBPβ protein expression, half‐life and ubiquitination. As expected, the ability of MIR503HG to suppress C/EBPβ was reversed (Figure 5E), and both the half‐life (Figure 5F) and ubiquitination levels (Figure 5G) of C/EBPβ normalised. These findings collectively suggest that MIR503HG promotes the ubiquitin‐mediated degradation of C/EBPβ by facilitating its binding to RNF43.

### 3.6 NETs reduce MIR503HG expression by triggering changes in DNA methylation

**FIGURE 6:**
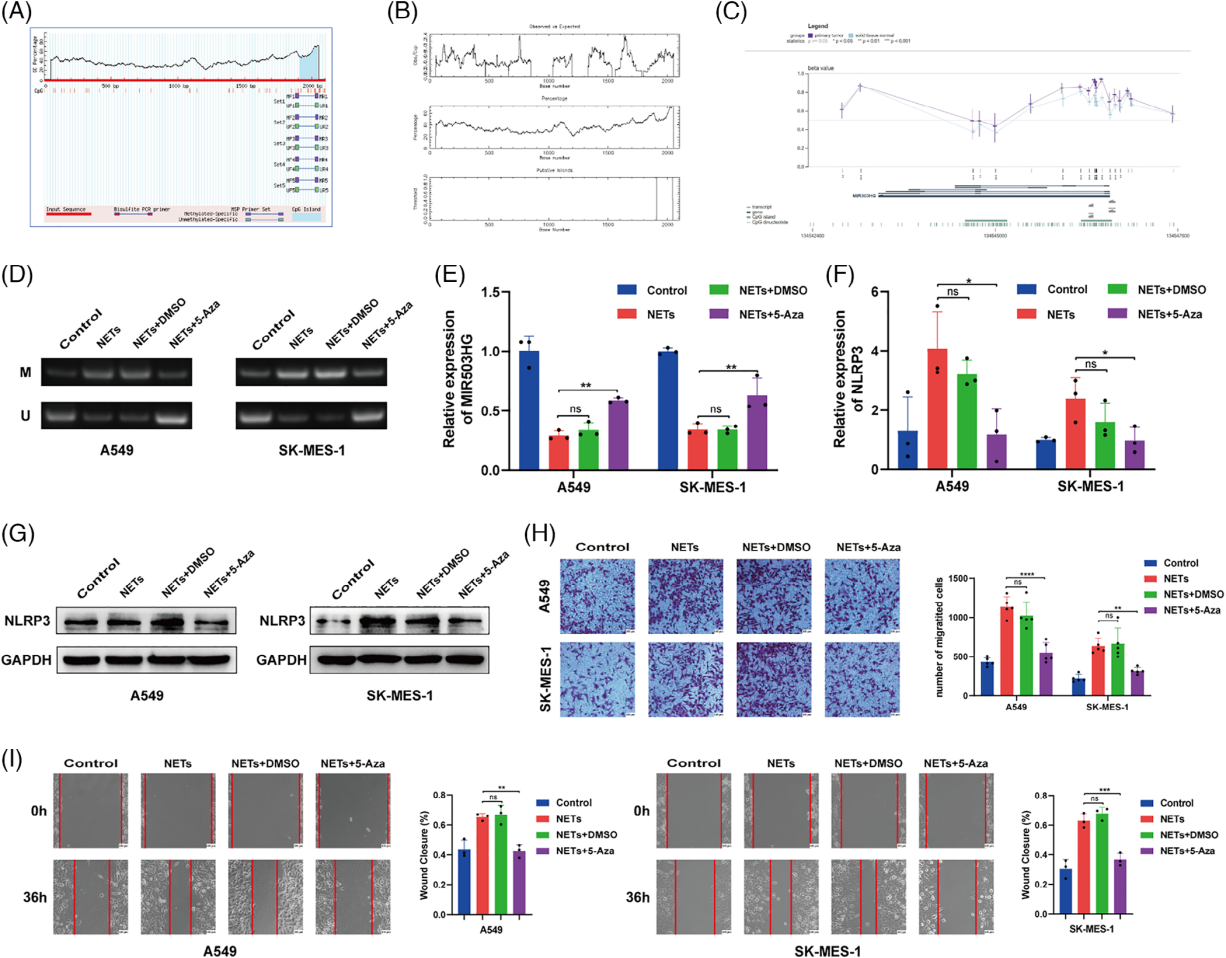
NETs reduce MIR503HG expression by triggering DNA methylation. CpG islands in the MIR503HG promoter were identified using MethPrimer (A) and EMBOSS (B). (C) MEXPRESS analysis indicated that the relative methylation level of the MIR503HG promoter was greater in primary tumours than in normal tissues. (D) MSP‐PCR was used to compare methylation levels in the MIR503HG promoter region following treatment of NSCLC cells with NETs and the methylation inhibitor 5‐Aza. M = methylated, U = unmethylated. (E) qRT‐PCR was used to measure MIR503HG expression after NETs formation and 5‐Aza treatment in NSCLC cells. NLRP3 mRNA and protein levels were assessed by RT‐qPCR (F) and Western blot (G) following the same treatments. Invasion (H) and wound‐healing (I) assays were conducted to evaluate NSCLC cell behaviour after NETs and 5‐Aza treatment; scale bar, 100 µm. Data in (E, F, H and I) are the mean ± SD. Data in (E, F and I) were derived from three independent experiments, while data in (H) were obtained from five independent experiments. The statistical method used for the data in (E, F, H and I) is one‐way analysis of variance (ANOVA). ns indicates no statistical significance, *p < .05, **p < .01, ***p < .001.

Previous studies have clarified the mechanism by which MIR503HG regulates NLRP3. We would like to further explore the molecular mechanism by which NETs inhibit MIR503HG. Given that DNA methylation is a primary regulator of lncRNA expression, we performed bioinformatic analyses, which revealed DNA hypermethylation at the MIR503HG transcription initiation site, as shown in both the MethPrimer (Figure 6A) and EMBOSS (Figure 6B) databases. Data from the MEXPRESS database indicated that methylation levels were elevated in primary NSCLC tissue compared with normal tissue (Figure 6C), suggesting a potential inverse relationship between methylation and the expression of MIR503HG. We hypothesised that NETs down‐regulate MIR503HG by inducing DNA methylation. This hypothesis was supported by MSP‐PCR, which revealed a significant increase in MIR503HG methylation following NETs treatment (Figure 6D). Notably, the inhibition of DNA methylation with 5‐azacytidine counteracted the effect of NETs on MIR503HG (Figure 6E) and significantly impacted NLRP3 mRNA (Figure 6F) and protein (Figure 6G) levels. Furthermore, pretreatment of cells with 5‐azacytidine before NETs exposure diminished the NETs‐induced invasion (Figure 6H) and migration (Figure 6I) of NSCLC cells. Our findings suggest that NETs suppress MIR503HG expression through DNA methylation, a mechanism critical for NETs‐mediated NSCLC metastasis.

## 4 DISCUSSION

NETs are crucial in tumour metastasis.^26^, ^27^ Our previous research demonstrated that NETs activate NLRP3 and facilitate NSCLC metastasis by suppressing MIR503HG. However, the specific mechanisms linking NETs, MIR503HG and NLRP3 were not fully elucidated. In this research, we revealed for the first time that DNA methylation and C/EBPβ are key regulators of the interactions among NETs, MIR503HG and NLRP3.

LncRNAs play pivotal roles in cancer progression by influencing transcription, translation and post‐translational modification.^28^, ^29^, ^30^ Previous research has indicated that MIR503HG suppresses NLRP3 expression by inhibiting its transcription. Based on these findings, we postulated that specific transcription factors are involved in the modulation of NLRP3 through MIR503HG. Transcription factors are regulatory proteins that bind to designated DNA sequences, controlling gene transcription. By integrating protein mass spectrometry of MIR503HG, NLRP3 transcription factor prediction and literature review, we identified seven transcription factors potentially involved in the regulation of NLRP3. Upon modulating these molecules, we found that knockdown of C/EBPβ, TRIM63, ZBTB10 and ZKSCAN1 significantly reduced the expression of NLRP3 mRNA and protein. Research has shown that ZBTB10 inhibits breast cancer metastasis.^31^ ZKSCAN1 is significantly involved in lymph node metastasis and vascular invasion in gastric cancer,^32^ while there are no relevant reports on TRIM63. Most notably, C/EBPβ not only promotes metastasis in various tumours but has also been documented in non‐cancer contexts where down‐regulation of C/EBPβ can suppress NLRP3 expression^20^, ^33^; however, the exact mechanisms remain unclear. In further experiments, we observed that C/EBPβ interacts with the NLRP3 promoter, enhancing its expression. These findings indicate that MIR503HG may repress NLRP3 by modulating C/EBPβ activity.

The overexpression of C/EBPβ is recognised as a significant factor in tumourigenesis. Recent studies have demonstrated that elevated C/EBPβ levels facilitate invasive metastasis across various cancers, such as gastric, pancreatic and breast cancer.^34^, ^35^, ^36^ Mechanistically, C/EBPβ promotes metastasis through the process of EMT.^35^ Thus, investigating the function of C/EBPβ in NSCLC metastasis, especially in patients with high NETs expression, is critical for advancing NSCLC treatment. Our findings reveal that C/EBPβ is markedly overexpressed in NSCLC tissues and facilitates metastasis in both in vivo and in vitro. Consistent with previous studies, the overexpression of C/EBPβ results in a decrease in E‐cadherin levels while simultaneously increasing the levels of N‐cadherin and vimentin, suggesting that C/EBPβ drives NSCLC metastasis through EMT. Additionally, we observed that MIR503HG interacts with C/EBPβ and inhibits its expression, supporting our hypothesis. This study is the first to show that MIR503HG reduces NLRP3 expression by down‐regulating C/EBPβ. However, the precise mechanism through which MIR503HG influences C/EBPβ expression remains to be fully elucidated.

Protein post‐translational modifications involve chemical changes to specific amino acid residues following protein synthesis, which regulate protein function, spatial distribution, structural conformation and molecular interactions with other biomolecules.^37^ Our findings indicate that while MIR503HG overexpression does not affect C/EBPβ transcription, it significantly reduces C/EBPβ protein levels. These results imply that MIR503HG influences C/EBPβ expression through post‐translational modifications. Ubiquitination, a complex and dynamic modification, is responsible for the degradation of more than 80% of intracellular proteins.^38^ Previous research has shown that MIR503HG impacts RNA‐binding proteins via the ubiquitination pathway. For example, Huang et al.^39^ revealed that MIR503HG modulates mesenchymal large‐cell lymphoma proliferation by affecting SUMRF2 ubiquitination. Similarly, in hepatocellular carcinoma, MIR503HG suppresses metastasis through the regulation of HNRNPA2B1 ubiquitination.^40^ In our study, proteasome inhibition with MG132 mitigated the MIR503HG‐induced reduction in C/EBPβ levels. Additionally, MIR503HG overexpression led to a decreased C/EBPβ half‐life and increased ubiquitination. Therefore, we conclude that MIR503HG inhibits C/EBPβ expression through the ubiquitination pathway.

Despite MIR503HG lacking ubiquitinating enzyme activity,^41^, ^42^ our protein mass spectrometry analysis identified RNF43 as a potential E3 ubiquitin ligase involved in MIR503HG‐mediated ubiquitination of C/EBPβ. RNF43 is known for its role in malignant tumour progression because of its E3 ubiquitin ligase activity.^43^, ^44^ In this research, we showed that RNF43 binds to C/EBPβ and facilitates its ubiquitin‐mediated degradation. Mechanistically, MIR503HG enhances the interaction between C/EBPβ and RNF43, thereby promoting C/EBPβ degradation. Previous research has highlighted the role of lncRNAs as scaffolds that facilitate protein‒protein interactions.^45^ For instance, Yuan et al.^23^ documented that LncSLC26A4‐AS1 inhibits thyroid metastasis by promoting the binding of DDX5 and the E3 ligase TRIM25. These findings suggest that RNF43 is crucial in the ubiquitination‐mediated degradation of C/EBPβ by MIR503HG. Furthermore, proteomic analysis of MIR503HG revealed other E3 ubiquitin ligases, such as TRIM4, PJA1 and TRIP12, which have been demonstrated to inhibit tumour progression in breast cancer and NSCLC.^46^, ^47^, ^48^ Notably, PJA1, similar to RNF43, plays a role in regulating the Wnt/β‐catenin signalling pathway. However, there is no explicit evidence suggesting their involvement in C/EBPβ regulation, which could be a potential area for future research.

Next, we explored the mechanism by which NETs inhibit MIR503HG expression. Growing evidence suggests that epigenetic modifications mediated by DNA methylation are essential in regulating the expression of LncRNAs and the related biological processes.^49^ For instance, Dong et al.^50^ demonstrated that DNA methylation facilitates the metastasis of oesophageal squamous cell carcinoma by down‐regulating Lnc00886 expression. Furthermore, multiple investigations have demonstrated that lncRNA expression is influenced by DNA methylation across various tumour types.^51^, ^52^, ^53^ Through bioinformatics analysis, we confirmed the existence of CpG island in the promoter region of MIR503HG. Our findings demonstrate that NETs repress MIR503HG expression and modulate downstream pathways and cellular functions by inducing changes in DNA methylation. In agreement with our results, Wang et al.^54^ also observed that the expression of MIR503HG in papillary renal cell carcinoma is regulated through DNA methylation. These findings suggest that NETs inhibit MIR503HG expression by promoting DNA methylation, indicating that targeting DNA methylation may be an effective strategy to prevent enhanced metastasis associated with NETs.

In summary, our findings have provided valuable insights into the molecular mechanisms by which NETs promote metastasis in NSCLC. A limitation of our study is that NETs do not fully replicate the in vivo environment, potentially introducing biases in understanding their functional mechanisms. Nevertheless, the results hold significant clinical relevance. Our research highlights the critical roles of C/EBPβ and DNA methylation in NETs‐induced NSCLC metastasis. These insights could pave the way for new therapeutic strategies targeting high NETs expression in NSCLC patients.

## 5 CONCLUSION

In the current investigation, we illustrated that C/EBPβ and DNA methylation are crucial in NETs‐induced NSCLC metastasis, which is correlated with poor outcomes. We observed that NETs inhibit MIR503HG expression through changes in promoter methylation. Furthermore, C/EBPβ, which acts as a regulatory factor for NLRP3, is regulated by MIR503HG. Notably, MIR503HG reduces C/EBPβ levels by increasing its interaction with RNF43, which leads to lower NLRP3 expression and consequently inhibits NSCLC metastasis.

## AUTHOR CONTRIBUTIONS

Xin Ye, Chen Fang and Weiwei Hong completed most of the experiments, data analysis and manuscript writing. Xiaoying Qian, Biao Yu and Bingbiao Zhou participated in animal experiments, immunofluorescence experiments, collection of human tissue samples and immunohistochemistry experiments. Xinyuan Yao, Dengying Chen and Chengsi Shu contributed to parts of the manuscript writing. Chuanhong Luo was involved in some of the RT‐qPCR and Western blotting experiments. Yong Wang and Yong Li designed the study and provided direction and guidance. All authors read and approved the final manuscript.

## CONFLICT OF INTEREST STATEMENT

The authors declare no conflicts of interest.

## ETHICS STATEMENT

This study involving research on animals or human tissues was reviewed and approved by the Ethics Committee of the Medical Innovation Center, First Affiliated Hospital of Nanchang University.

## Supporting information



Supporting Information

## Data Availability

The data that support the findings of this study are available from the corresponding author upon reasonable request.
