# Supplementary material for: LncRNA MIR503HG regulates NETs‐mediated NLRP3 inflammasome activation and NSCLC metastasis by enhancing the ubiquitination of C/EBPβ
Source: Clin Transl Med. 2025 Jun 9;15(6):e70342. doi: 10.1002/ctm2.70342 (PMC12148952; doi:10.1002/ctm2.70342)
Supplement: Supplementary file 1 — Supporting Information [file CTM2-15-e70342-s001.docx]

**Supplementary Figures, Figure Legends and Tables**

**
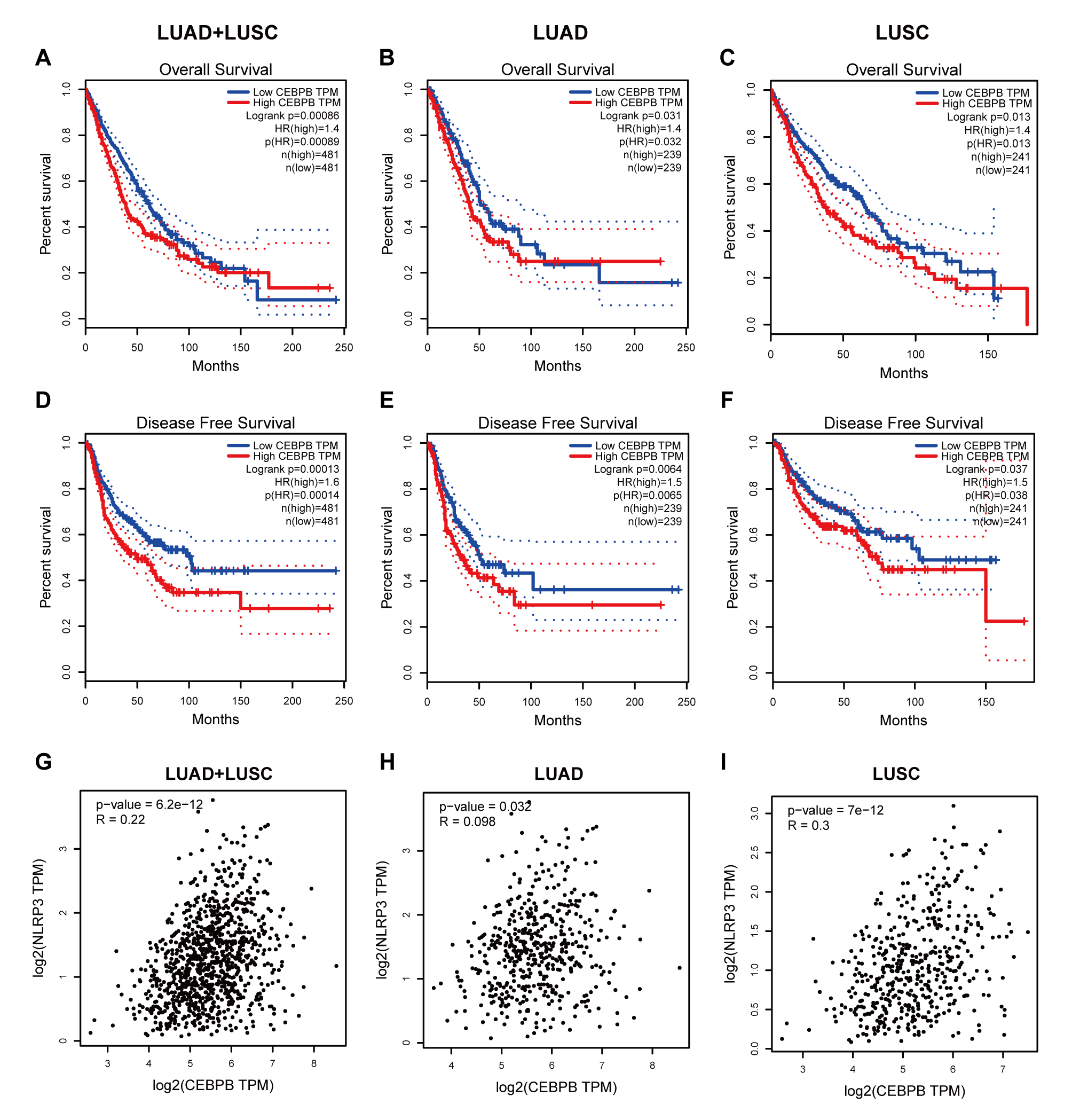
**

**Supplementary Figure S1 (related to Figure 1)** C/EBPβ is linked to NLRP3 expression and correlates with poor prognosis in NSCLC patients. The associations between C/EBPβ expression and survival outcomes of NSCLC patients were analyzed using the GEPIA online tool on the basis of data from the TCGA database. (A) Relationship between C/EBPβ expression and overall survival (OS) in NSCLC patients. (B) Association between C/EBPβ expression and OS in lung adenocarcinoma (LUAD) patients. (C) The correlation between C/EBPβ expression and overall OS in lung squamous cell carcinoma (LUSC) patients. (D) Link between C/EBPβ expression and disease-free survival (DFS) in NSCLC patients. (E) Relationship between C/EBPβ expression and DFS in LUAD patients. (F) Association between C/EBPβ expression and DFS in LUSC patients. (G and H) The correlation between C/EBPβ and NLRP3 gene expression was analyzed in tumor tissue samples from NSCLC patients using the GEPIA website, where R > 0 indicates a positive correlation and R < 0 indicates a negative correlation.


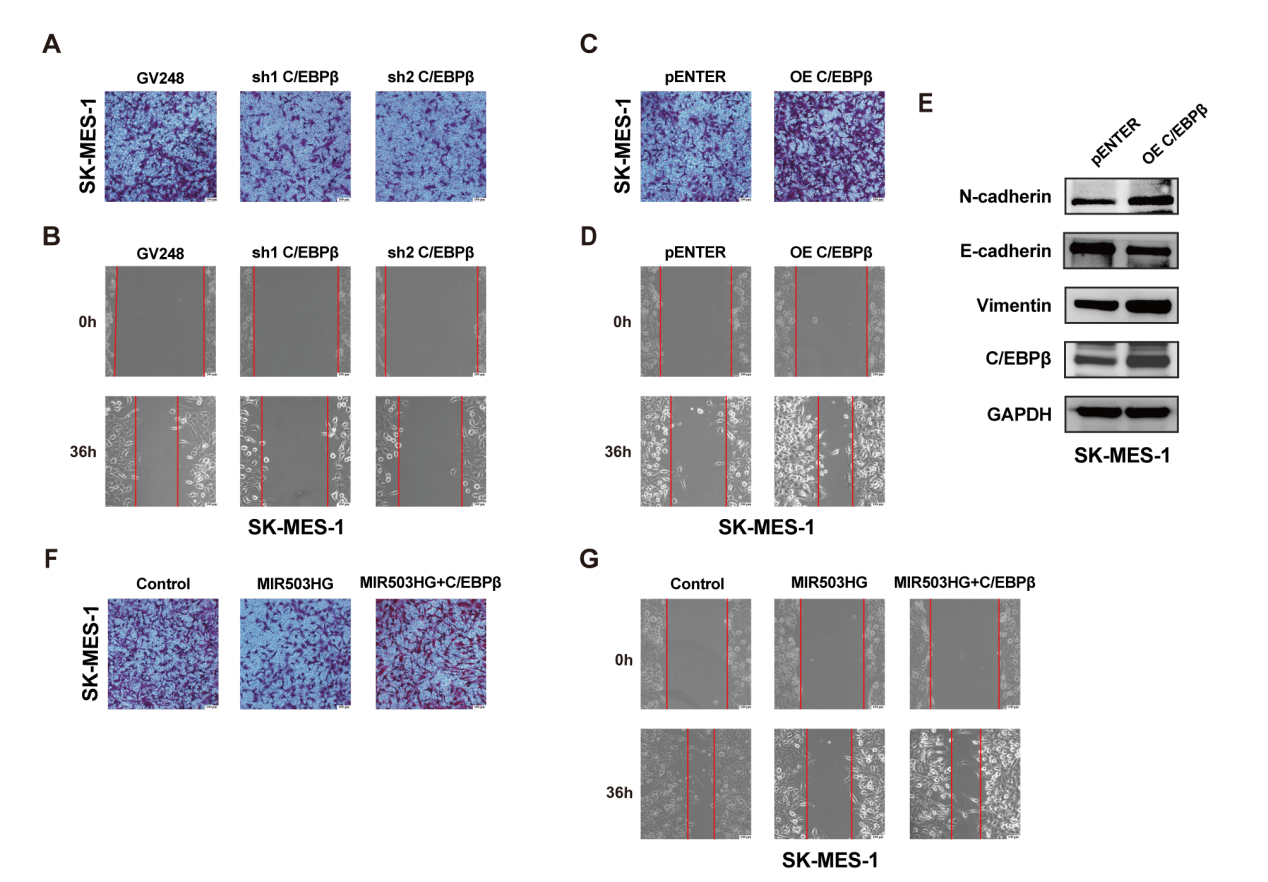


**Supplementary Figure S2 (related to Figure 2)** C/EBPβ promotes the invasion and metastasis of SK-MES-1 cells. The invasive and migratory capabilities of SK-MES-1 cells following C/EBPβ downregulation were assessed using transwell (A) and wound-healing (B) assays. The impact of C/EBPβ overexpression on the invasion (C) and migration (D) of SK-MES-1 cells was also evaluated. (E) Western blot analysis was performed to examine the expression of EMT markers (N-cadherin, E-cadherin, and vimentin) after C/EBPβ overexpression in SK-MES-1 cells. (F and G) The effects of MIR503HG overexpression combined with C/EBPβ activation on the invasive and migratory properties of SK-MES-1 cells were investigated.


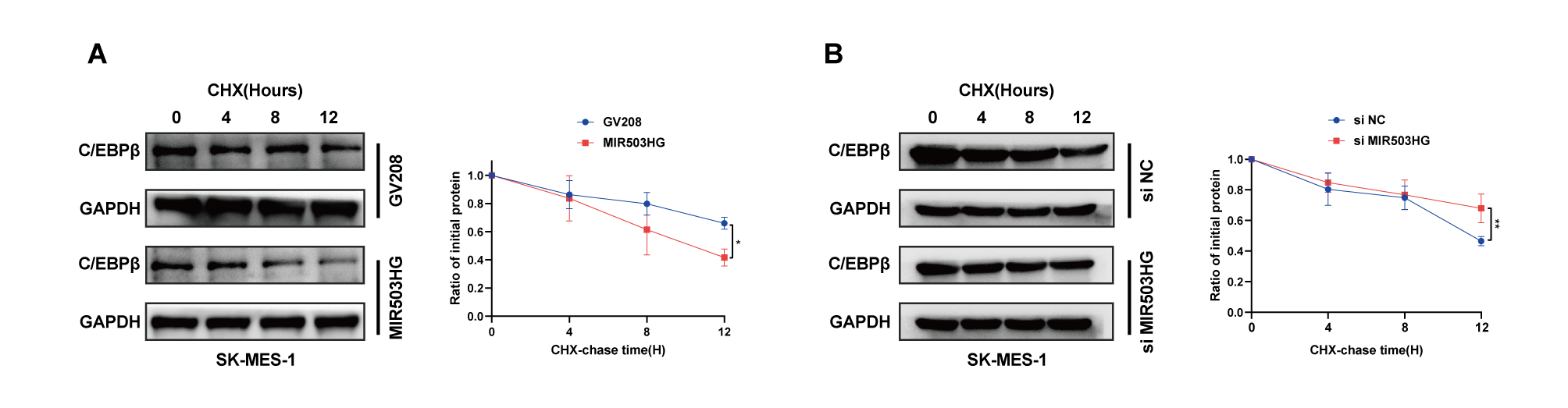


**Supplementary Figure S3** **(related to Figure 3)** MIR503HG reduces the half-life of C/EBPβ. Western blot analysis of C/EBPβ protein levels was conducted after treatment with cycloheximide (CHX) following either overexpression (A) or knockdown (B) of MIR503HG. Data in (A-B) are the mean ± SD. of three independent experiments. The statistical method used for the data in (A-B) is two-way analysis of variance (ANOVA). **P* < 0.05, ***P* < 0.01.


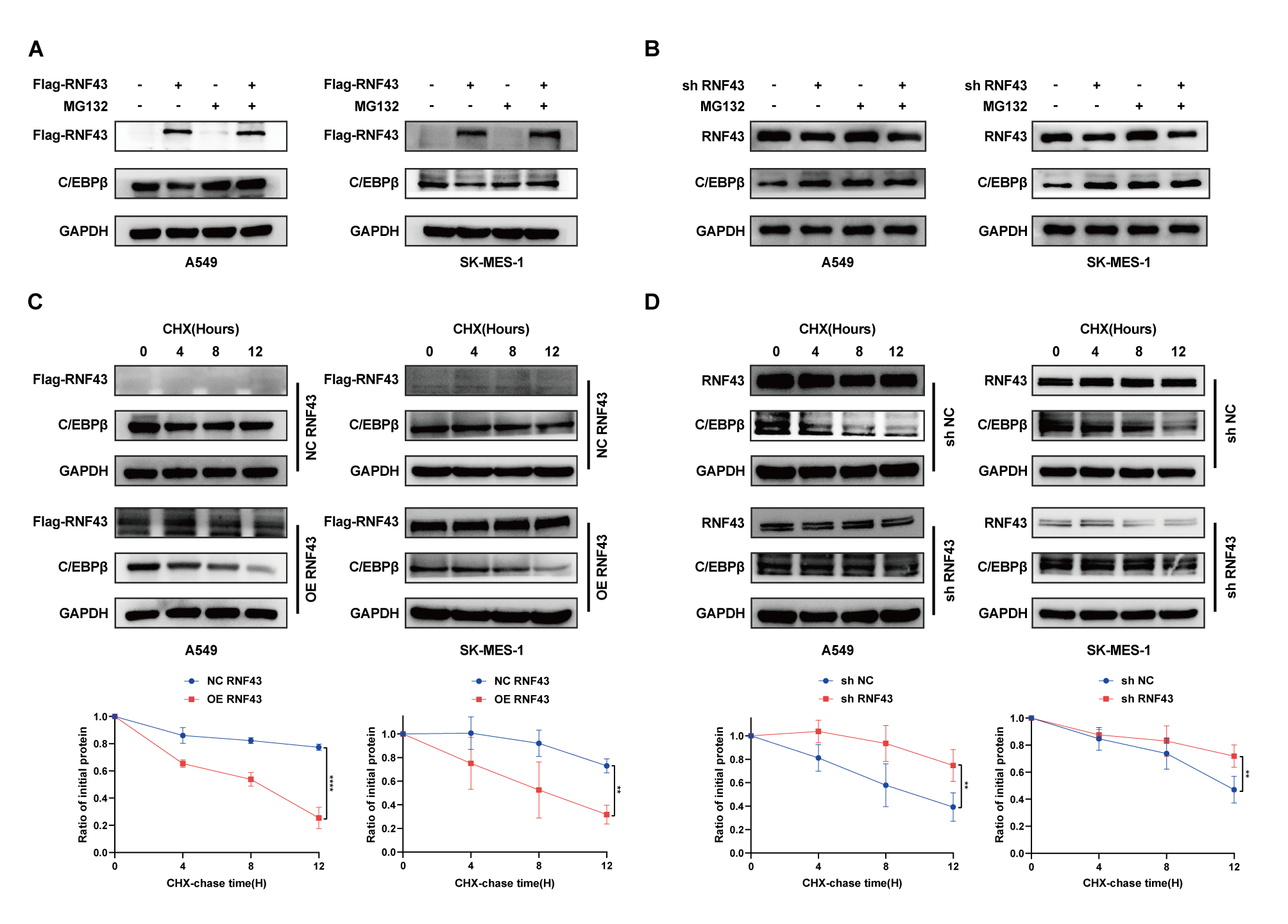


**Supplementary Figure S4** **(related to Figure 4)** RNF43 enhances C/EBPβ degradation via the ubiquitination pathway. Western blot analysis was used to measure C/EBPβ protein levels in cells with MIR503HG knockdown or overexpression following treatment with MG132 (A and B) or cycloheximide (CHX) (C and D). Data in (C-D) are the mean ± SD. of three independent experiments. The statistical method used for the data in (C-D) is two-way analysis of variance (ANOVA). ***P* < 0.01, *****P* < 0.0001.

**Table S1. Gene knockdown target sequence information.**

| **Gene (human)** | **Target sequence information** |
| --- | --- |
| si-MIR503HG#1 | CCUCUCCCACCAUUUCUUUTT |
| si-MIR503HG#2 | GACAAGAACUAAAGUGGAATT |
| si-GTF3C2# | GUAGAGAUGUCAUUACCUATT |
| si-TRIM63# | GCCUGGAGAUGUUUACCAAGC |
| si-ZBTB10# | GGCCAAAUGAGAACUCUUATT |
| si-INSM1# | CACAAGUACUUCGAACGCATT |
| si-C/EBPβ# | GCCCUGAGUAAUCGCUUAATT |
| si-EHF# | GCCAGUGGCAUGAAAUUCATT |
| si-ZKSCAN1# | GGACCUGAGAUGCUCGCAATT |
| sh-C/EBPβ-1 sense | CCCGTGGTGTTATTTAAAGAA |
| sh-C/EBPβ-1 antisense | TTCTTTAAATAACACCACGGG |
| sh-C/EBPβ-2 sense | GCTGCGCGCTTACCTCGGCTA |
| sh-C/EBPβ-2 antisense | TAGCCGAGGTAAGCGCGCAGC |
| sh-RNF43-1 sense | CCACCTCCAATCCACCTCACAT |
| sh-RNF43-1 antisense | ATGTGAGGTGGATTGGAGGTGG |
| sh-RNF43-2 sense | CTCCACCTCATTCGCCAGCATT |
| sh-RNF43-2 antisense | AATGCTGGCGAATGAGGTGGAG |

**Table S2. Solution preparation information.**

| **Solution** | **Solution preparation** |
| --- | --- |
| Buffer I | Urea, 0.5M Na₂HPO₄/NaH₂PO₄ (pH=8.0), 1M Tris-Cl (pH=8.0), 14.4M β-ME, and 1M imidazole. |
| Buffer II | Urea, 0.5M Na₂HPO₄/NaH₂PO₄ (pH=6.3), 1M Tris-Cl (pH=6.3), 14.4M β-ME, and 1M imidazole. |

**Table S3. Primer Sequence**

| **Gene (human)** | **Forward（5′-3′）** | **Reverse（5′-3′）** |
| --- | --- | --- |
| MIR503HG | CCAGCCAGCCTTCCTGAAAG | ACTGGAGATGCTGGATGCCT |
| NLRP3 | GATCTTCGCTGCGATCAACAG | CGTGCATTATCTGAACCCCAC |
| GAPDH | TCTGGAAAGCTGTGGCGTGA | AGCTCTGGGATGACCTTGCC |
| C/EBPβ | CGCGCTTACCTCGGCTACCA | GCGCCGGATCTTGTACTCGTC |
| MIR503HG-M | TTATTCGTAGGTATTTTTAGATCGA | CCTAATAACAAAAACACGCGTC |
| MIR503HG-U | TTATTTGTAGGTATTTTTAGATTGA | ACACCTAATAACAAAAACACACATC |
| NLRP3-CHIP | GAACATGGGGAGTGGAGG | GCACTACCGTGAGAGGG |

**Table S4. Antibodies and agents of experiments.**

| **Product** | **No. of Catalogue** | **Supplier** |
| --- | --- | --- |
| NLRP3 antibody | 19771-1-AP | Proteintech |
| GAPDH antibody | 60004-1-Ig | Proteintech |
| E-cadherin antibody | 20874-1-AP | Proteintech |
| N-cadherin antibody | ab76011 | Abcam |
| Vimentin antibody | ab92547 | Abcam |
| C/EBPβ antibody | 23431-1-AP | Proteintech |
| Flag antibody | F1804 | Merck |
| His antibody | 66005-1-lg | Proteintech |
| HA antibody | 66006-2-lg | Proteintech |
| GST antibody | 66001-2-Ig | Proteintech |
